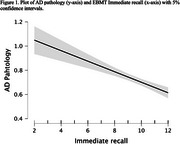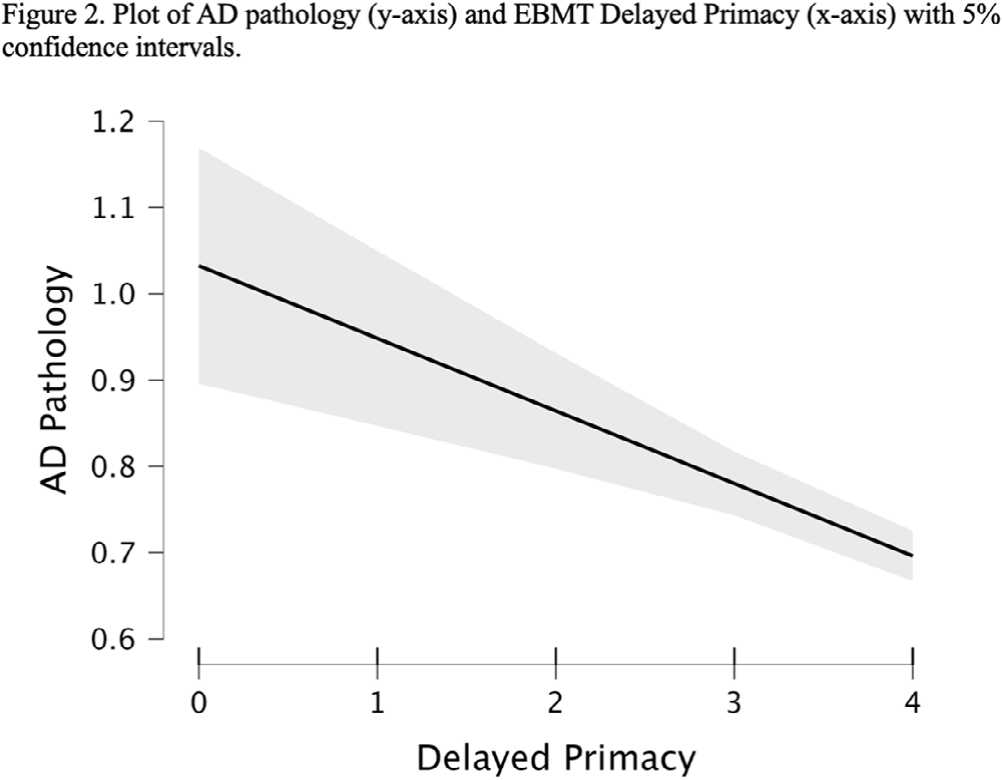# Short story recall and prediction of *postmortem* Alzheimer's pathology

**DOI:** 10.1002/alz70857_097981

**Published:** 2025-12-24

**Authors:** Davide Bruno, Ainara Jauregi Zinkunegi, Kimberly D Mueller, Melissa Lamar

**Affiliations:** ^1^ Liverpool John Moores University, Liverpool, United Kingdom; ^2^ Department of Communication Sciences and Disorders, University of Wisconsin‐Madison, Madison, WI, USA; ^3^ Department of Psychiatry and Behavioral Sciences, Rush University Medical Center, Chicago, IL, USA

## Abstract

**Background:**

The East Boston memory test (EBMT) is a short story recall test that compares favorably to longer story recall tests like the logical memory test. However, little is known as to how well EBMT predicts *postmortem* Alzheimer's disease (AD) pathology, and whether item‐based serial position analysis is applicable to it for this purpose.

**Methods:**

Data from 1699 individuals participating in three different Rush University study cohorts were examined (age = 79.7, SD = 7.1). The majority of the sample was comprised of women (69%). All participants completed the test in English and were free of dementia at baseline.

Analyses were carried out with Bayesian and Frequentist statistics. Regression analyses were applied to predict overall *postmortem* AD pathology longitudinally from baseline. Predictors were immediate EBMT recall, delayed EBMT recall, and serial position metrics, i.e., the order in which story items were learned. Control variables were age at baseline, gender, years of education, time between baseline and death, and *APOE* e4 status.

**Results:**

Inspection of q‐q plots suggested the data were suitable for linear regressions. Results from the Bayesian analyses showed that the best fitting model included two predictors: immediate recall (BFinclusion = 4069) and delayed primacy recall (BFinclusion = 9), i.e., remembering the beginning of the story after a delay. The two predictors did not meaningfully interact. Frequentist tests confirmed that including either immediate recall (AIC = 2884; Figure 1) or delayed primacy (AIC = 2896; Figure 2) in the models improved fit over control variables (AIC = 2915). Further tests indicated that immediate and delayed primacy recalls predicted *postmortem* neuritic plaques and neurofibrillary tangles burdens, but not diffuse plaques burden.

**Conclusion:**

In summary, EBMT is suitable to use for early prediction of AD pathology from a cognitively healthy baseline, despite its brevity. Additionally, immediate recall and delayed primacy performance are independent contributors to the prediction of post‐mortem AD‐related neuropathology.